# Current News and Opinion

**Published:** 1882-04

**Authors:** 


### CURRENT NEWS AND OPINION.

Dr. S. L. Mason died in Brooklyn on February 12th, aged seventy-nine years.

Professor Duchek, of the University of Vienna, died March 2d, aged fifty-eight.

Dr. John Osborn, of this city, died on March 24th in the sixty-fourth year of his age.

The University of Wurzburg, Bavaria, will celebrate its 300th anniversary August 1–3 of this year.

A congress of Greek physicians and naturalists from all parts of the world met in Athens on April 18th.

A case of syphilis communicated by skin grafting is reported by Dr. Denhel in the *Gazette Méd. de Paris.*

Dr. L. A. McMurtry has been appointed Professor of Anatomy in the Kentucky School of Medicine.

Two journals which every progressive practitioner needs: The Independent Practitioner and the *Index Medicus.*

A home for poor consumptives is to be built in this city. It will be under the care of the Sisters of St. Francis.

ANOTHER micrococcus has been discovered. Neisser found it in trachoma. The good work goes bravely on!

THE NEXT meeting of the International Medical Congress will be held in Copenhagen in 1884. Professor Panum will preside.

THE DENTISTS have been at it again. Two deaths from the inhalation of chloroform in the dentist's chair are reported from Chicago.

PROFESSOR JOSEPH PANCOAST, the distinguished surgeon and anatomist, died in Philadelphia, on March 7th, aged seventy-seven years.

PROFESSOR OSCAR SIMON, Professor of Dermatology at the University of Breslau, Germany, died in that city on March 2d, aged thirty-eight.

PROFESSOR J. W. UNDERHILL, of Cincinnati, one of our collaborators, has been seriously ill. He is now, we are pleased to learn, on the road to recovery.

DR. ROBERT BRIDGES, Emeritus Professor of Chemistry in the Pennsylvania College of Pharmacy, died in Philadelphia, February 23d, in the seventy-sixth year of his age.

THE ANNUAL commencement of the Dental Department of the University of Pennsylvania was held March 15th. The degree of D.D.S. was conferred on forty-one candidates.

DR. WM. C. JARVIS has been appointed Lecturer on Laryngology in the University of the City of New York, in place of Professor Elsberg, whose resignation was announced in our last issue.

A NEW danger in anæsthesia consists in a "quid" of tobacco becoming lodged in the larynx. A case where this accident produced death in chloroform anæsthesia has been recently reported.

THE AMERICAN SPECIALIST has been discontinued, and *The Medical Register*, devoted to medical bibliography, takes its place. P. Blakiston, Son & Co., Philadelphia, are the publishers. $1 per annum.

DR. EDWARD WIGGLESWORTH gives the following diet list for out-patients at the Boston City Hospital: *Do not give* tea or coffee, cake or candy, new hot bread or pastry, nuts or fried meats, pork, pickles, lobsters or cheese. *Feed up well* on fresh meats, eggs, milk, bread, butter, and oatmeal.

THE UNIVERSITY OF CALIFORNIA has just completed the addition of a Department of Dentistry to its organization, with a corps of professors and teachers which ought to place the department on as high a level as the best Eastern schools. The regular term of instruction will commence on the 1st of June and continue five months. Dr. S. W. Dennis is Dean of the Faculty and Professor of Operative Dentistry and Histology.

Dr. R. S. Sutton has been travelling among the medical schools of the continent of Europe. He says: "In my judgment Halle is the best place for young Americans to study. It is cheap, clean, and free from all the attractions, such as large cafés and theatres, so very detrimental to young men and so very abundant in the large cities. The German here is also pure, and the teaching is in method simply faultless. If I had a son to educate, this is the first place I have found where I would be willing to place him."

